# Quantifying biochemical reaction rates from static population variability within incompletely observed complex networks

**DOI:** 10.1371/journal.pcbi.1010183

**Published:** 2022-06-22

**Authors:** Timon Wittenstein, Nava Leibovich, Andreas Hilfinger

**Affiliations:** 1 Department of Physics, University of Toronto, Ontario, Canada; 2 Department of Physics, Johannes Gutenberg University Mainz, Mainz, Germany; 3 Department of Mathematics, University of Toronto, Toronto, Ontario, Canada; 4 Department of Cell & Systems Biology, University of Toronto, Toronto, Ontario, Canada; 5 Department of Chemical & Physical Sciences, University of Toronto Mississauga, Mississauga, Ontario, Canada; Pázmány Péter Catholic University: Pazmany Peter Katolikus Egyetem, HUNGARY

## Abstract

Quantifying biochemical reaction rates within complex cellular processes remains a key challenge of systems biology even as high-throughput single-cell data have become available to characterize snapshots of population variability. That is because complex systems with stochastic and non-linear interactions are difficult to analyze when not all components can be observed simultaneously and systems cannot be followed over time. Instead of using descriptive statistical models, we show that incompletely specified mechanistic models can be used to translate qualitative knowledge of interactions into reaction rate functions from covariability data between pairs of components. This promises to turn a globally intractable problem into a sequence of solvable inference problems to quantify complex interaction networks from incomplete snapshots of their stochastic fluctuations.

## Author summary

Statistical models are the dominant tool to interpret co-variability of molecular components in cellular processes because they can be formulated for a subset of components while leaving the full complexity of the processes unspecified. Their drawback lies in the difficulty of translating statistical associations into causal interactions. In contrast, complete mechanistic models of biochemical reaction networks are a powerful tool to describe physical interactions, but often necessitate making a large number of assumptions such that each individual assumption is only marginally tested in global model comparisons with data. We introduce a novel inference method that combines the power of both approaches by exploiting testable predictions for only partially specified mechanistic models. We present numerical proof-of-principle examples in which we reconstruct biochemical reaction rates from partial observations of variability within simulated biochemical reaction networks in the absence of cell division. In contrast to existing approaches, our algorithm does not require perturbations, temporal information, observing all components within a complex network, or complete model knowledge. Its key ingredients are partial knowledge of qualitative network interactions paired with high precision probability distributions to quantify stochastic fluctuations within biochemical reaction networks.

## Introduction

Quantifying interactions between components within a complex network from snapshots of their activity is a challenge common to many areas of science. For example, understanding cellular processes requires quantifying biochemical reaction rates between molecules while typical high-throughput methods such as single-cell sequencing [1, 2], flow cytometry [3–5], or a combination thereof [6] generate static population snapshots of a subset of cellular components.

Covariability of components within cellular processes is typically analyzed using statistical associations [7–13] because accurate mechanistic modelling of biochemical reactions is challenging for complex systems due to the large number of unknown parameters and interactions [14]. However, molecular abundances are set by underlying physical interactions that affect each component’s rate of production and degradation rather than its instantaneous concentration. How molecular components affect each other is then difficult to infer from statistical associations [15], especially in the absence of perturbation experiments [16, 17]. For example, even perfectly linear *rate* dependencies will lead to non-linear statistical relations between observed *values* of cellular components.

**Fig 1:**
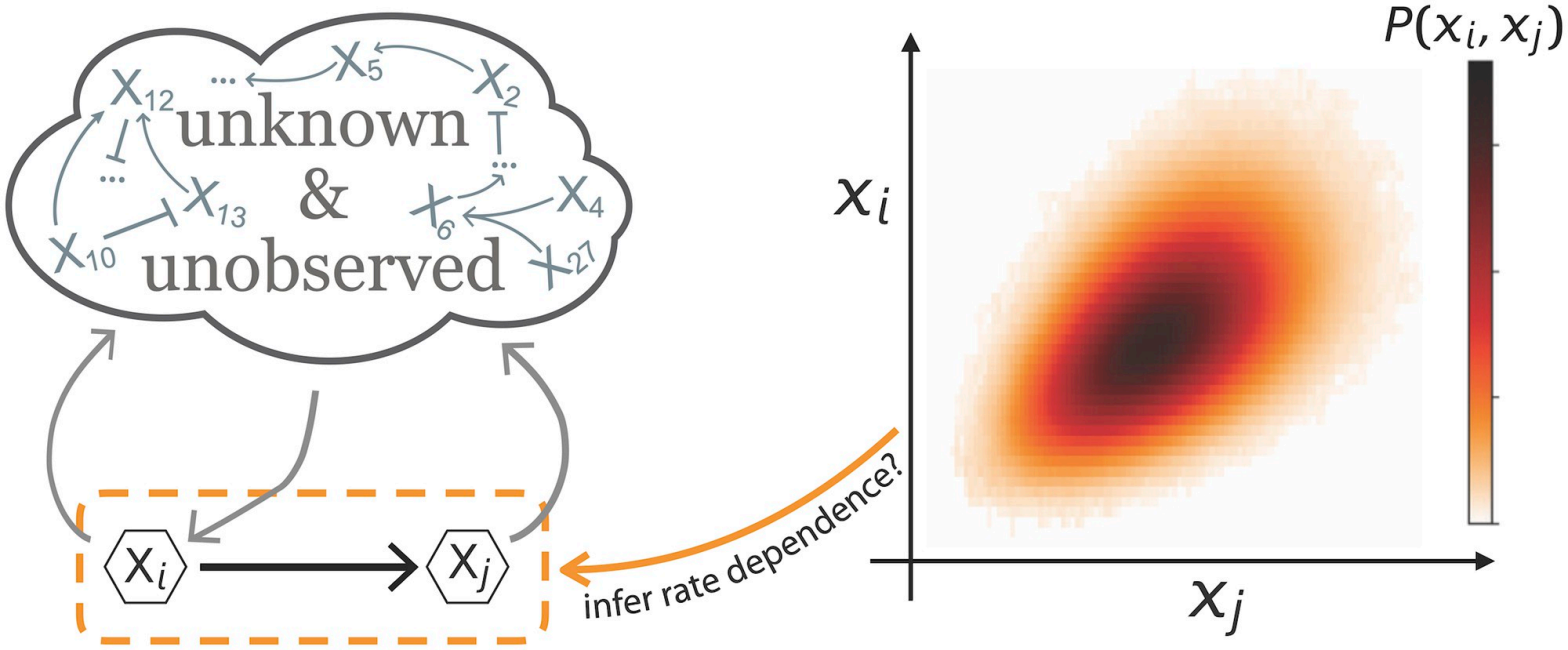
Our goal: Determining how one component affects the production rate of another from observing their joint probability distribution in a sea of unobserved components with unknown dynamics. The joint probability distribution between two components *P*(*x*_*i*_, *x*_*j*_) varies enormously between systems even for identical regulation of X_*i*_ through X_*j*_. However, previous work has shown that all systems with a given interaction between the two components satisfy invariant probability flux balance relations [18]. Here, we demonstrate that such relations can translate qualitative local network knowledge into quantitative rate functions using only the observed joint probability distributions between X_*i*_ and X_*j*_ even when the dynamics within the rest of the network is unobserved and completely unknown.

Mechanistic models that quantify causal interactions between components would thus be preferable to describe biochemical reaction networks. However, constructing complete mechanistic models requires describing every interaction in a system. For complex cellular processes, often only some of the mechanistic details of molecular interactions will be known in detail, some aspects will have to be estimated from comparable systems reported in the literature, and some will have to be postulated because of a lack of direct experimental evidence. Here, we introduce a novel data analysis approach to deduce mechanistic rate dependencies *one interaction at a time* using incompletely specified mechanistic models, see Fig 1. Its key advantage is that only the interactions of the local network need to be specified and the mechanistic details of regulation or degradation of all other components within the network do not need to be modelled. This approach exploits a local qualitative understanding of network interactions through probability balance equations [18] that must be satisfied as long as we know how one component is made and degraded. In contrast to existing work [13, 19, 20], our approach does not require temporal information, experimental perturbations, or complete observation of all components within an interaction network.

We present numerical evidence for four distinct network topologies in which we successfully infer how one component affects the production rate of another from their observed joint probability distribution without without making any assumptions about the dynamics of the non-observed components. This proof-of-principle across four different network dynamics illustrates how qualitative knowledge of local network interactions can be translated into quantitative rate functions using only static snapshots of naturally occurring population variability.

### Background theory

Describing the dynamics of some components within a complex interaction network in which the interactions between many components are unknown may seem impossible. However, we can trivially do so as long as we are content with describing one component’s dynamics in terms of components directly affecting it. The actual dynamics are fundamentally indeterminable for incomplete models but if components of interest are experimentally measurable their empirically observed covariability can be used to close the problem and constrain interaction rates as described below.

We follow the previously established approach [18] to characterize “local” system dynamics within a completely general complex reaction network with probabilistic events

$$\begin{array}{c} x\overset{r_{k}\left(x\right)}{\to }x+d_{k}\;k=1,2,3,\ldots \end{array}$$

where the state vector **x** = (*x*_1_, *x*_2_, *x*_3_, …) of abundances can be arbitrarily high-dimensional, the *k*^*th*^ reaction changes levels of component X_*i*_ by *d*_*ki*_, and the reaction rates *r*_*k*_(**x**) are arbitrarily non-linear functions of the state vector. This notation is motivated by biochemical reaction networks but many areas of science encounter stochastic systems whose dynamics are determined by the corresponding general chemical master equation

$$\begin{array}{c} \frac{dP(x,t)}{dt}\;=\;\sum_{k}[r_{k}\left(x\;-\;d_{k}\right)P\left(x\;-\;d_{k},t\right)\;-\;r_{k}\left(x\right)P\left(x,t\right)] \end{array}$$ (1)

where *P*(**x**, *t*) denotes the probability of the system to be in state **x** at some time *t*. Directly solving Eq (1) is impractical for many complex cellular processes of interest because, generally, not all reactions rates are known, and because non-linear rates in complex systems generally render them analytically intractable due to moment closure problems [21, 22], although exceptions exist [23].

However, even when many details of a system are unknown, any given molecular component X_*i*_ that reaches a time-independent stationary state with probability distribution *P*_*ss*_(*x*_*i*_) must satisfy

$$\begin{array}{c} 0=\sum_{k}[\left\langle r_{k}\left(x\right)|x_{i}=m-d_{ki}\right\rangle P_{ss}\left(x_{i}=m-d_{ki}\right)-\left\langle r_{k}\left(x\right)|x_{i}=m\right\rangle P_{ss}\left(x_{i}=m\right)]\\ \forall m\in \text{I}\;{\text{N}}_{0} \end{array}$$ (2)

which follows from simple summation of Eq (1) over all other variables and has been derived and discussed previously [18, 24]. Here, and throughout the article, angular brackets denote averages over the stationary state distribution.

In this paper, we demonstrate that Eq (2) can be exploited to infer rate functions even when we know nothing about the dynamics of all other components X_*j*_ for *j* ≠ *i* such that conditional rates cannot be predicted from incompletely specified models. Note, Eq (2) is not an approximate coarse-graining but corresponds to an exact balance relation for any variable in a larger complex system at stationarity. This stationarity assumption allows for a broad class of biological systems to be analyzed: Eq (2) requires that the joint probability distribution of interest does not change over time, which can be verified experimentally from population snapshots taken at different time-points. Thus, the only dynamics excluded from our analysis is transient behaviour such that stationary probability distributions are not accessible from experimental data. Even explicitly time-varying systems that technically never reach a stationary state, such as deterministic oscillations, satisfy Eq (2) when considering their time-averaged probability distributions and rates [18]. Although care has to be taken when comparing such time-averages with population averages in growing populations [25].

Next, we present results to show that stationary state probability distributions contain enough information to reconstruct an entirely unknown rate function in an otherwise unknown network of interactions. This is in contrast to an analysis of deterministic steady-states which only give one balance equation for each variable of interest and do not contain enough information to reconstruct a general rate function that is not parameterized by a single parameter.

## Results

When the rates of all reactions directly changing X_*i*_-levels are known, Eq (2) represents a self-consistency check that must be satisfied by the observed joint probability distribution between X_*i*_ and all variables directly affecting those rates. Next, we show how this relation can be “inverted” to determine rate functions from observed probability distributions.

While Eq (2) must provably hold for all stationary states, any empirically observed distribution will exhibit sampling errors which can have significant effects. For example, any real experiment will have some maximum value *m*_max_ for which X_*i*_ is observed and thus Eq (2) will clearly be violated for unbounded systems because Eq (2) cannot balance when *m* = *m*_max_ due to the lack of sampling of the rarest states. Inverting the equation system Eq (2) to identify the functional dependencies of *r*_*k*_(**x**) thus requires minimizing deviations from the predicted relations. In general, minimizing the sum of squared differences remains an underdetermined problem if we treat each value of the rate function as an independent unknown. However, under the assumption that biochemical rate functions are sufficiently smooth the problem can be solved by limiting the variability of the rate functions across neighbouring states (Materials & methods).

**Fig 2:**
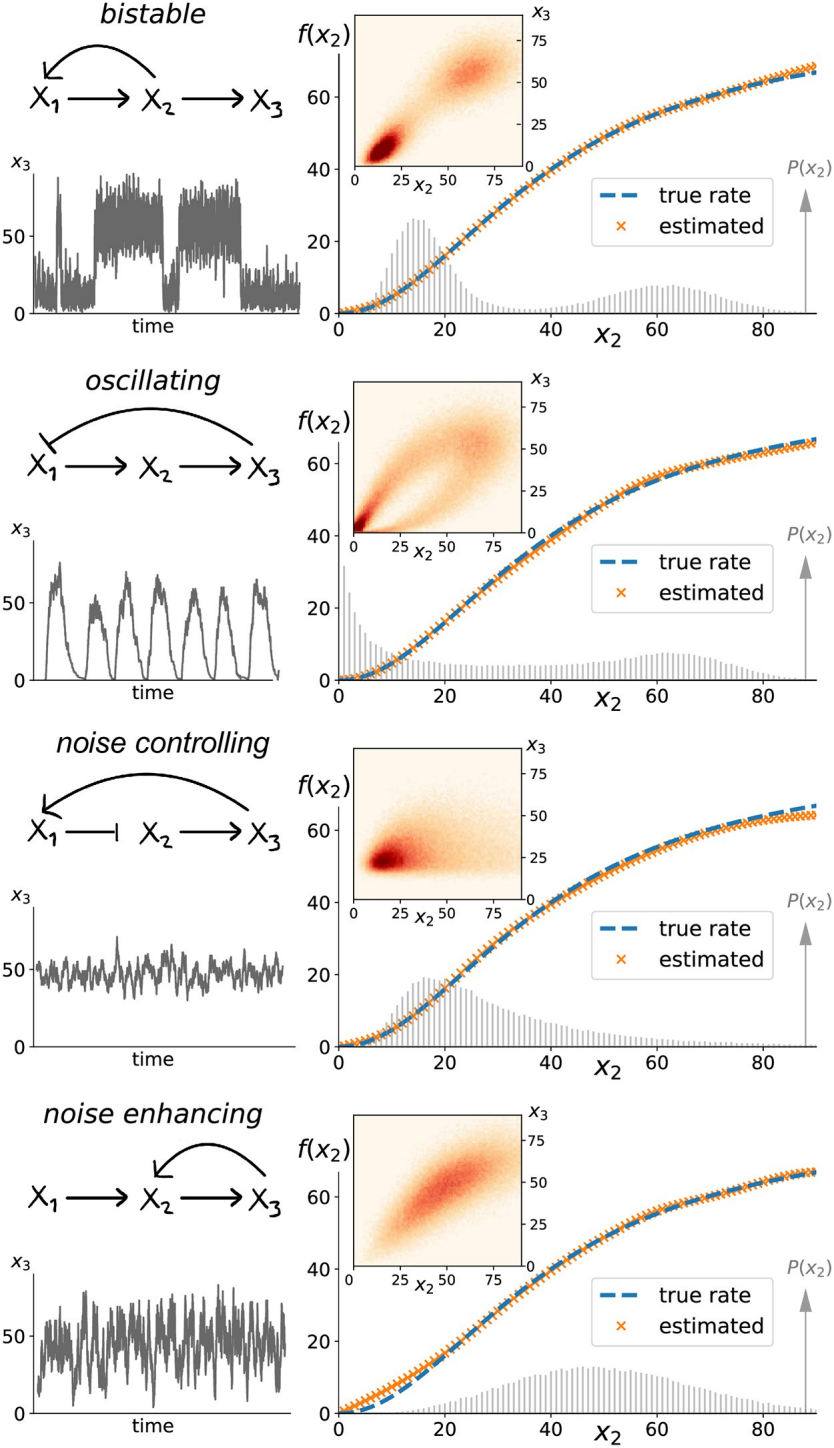
Probability flux balances can determine biochemical rates regardless of global network dynamics. Fixing how the production rate *f*(*x*_2_) of X_3_ depends on X_2_-levels, we considered four different global network topologies within the class defined by Eq (3), that exhibit diverse system dynamics and variability in X_3_ (left column). The insets of the right column depict numerically observed joint distributions *P*(*x*_2_, *x*_3_) corresponding to 100,000 independent snapshots. Although probability distributions differed greatly between the four systems, Eq (4) could identify the functional dependence of the production rate of X_3_ based on the numerical convex optimization algorithm detailed in the Materials & Methods. We find near perfect agreement between the inferred rate (orange crosses) and the true rate function (dashed blue line) regardless of a system’s global dynamics. This inference of *f*(*x*_2_) does not utilize any temporal information, its only input is the stationary joint probability distribution between the two components of interest. It relies on observing fluctuations across a wide range of X_2_-states as illustrated by the shaded probability distribution *P*(*x*_2_) with deviations occurring where X_2_ was rarely or never observed. While the degradation rate of X_3_ was assumed to be known, no information about how its production rate depends on X_2_, or the dynamics of X_1_, X_2_ was used.

To demonstrate how rate functions can be successfully inferred from partial observations of some components within a larger network we consider four different example networks that exhibited oscillations, bistability, fluctuation control, and noise enhancing feedback. Such markedly different global dynamics was already achievable with non-linear three-component feedback networks while conserving the reaction rates for one of the components, see Fig 2 (left panels). We thus present the performance of our algorithm when applied to simulation data from those simple systems. But the algorithm can equally be applied to much larger systems with hundreds of variables as long as the reactions directly affecting X_*i*_ are qualitatively known.

### Numerical proof-of-principle examples

The conserved part of our test systems that we want to reconstruct from simulation data corresponds to a stereotypical biochemical reaction rate in cells. In particular, we specified that the production of component X_3_ is affected by X_2_ through a Hill-type function *f*(*x*_2_) and X_3_ molecules are degraded independently leading to the following class of reaction systems

$$\begin{array}{c} \underset{\text{conserved}\;\text{part}}{\underset{︸}{\begin{array}{ccc} x_{3} & \overset{f\left(x_{2}\right)}{\to } & x_{3}+1\\ x_{3} & \overset{x_{3}/\tau_{3}}{\to } & x_{3}-1 \end{array}}}+\underset{\text{various}\;\text{feedback}\;\text{dynamics}}{\underset{︸}{\left[\begin{array}{c} {\text{X}}_{1}{\text{,X}}_{2}\\ \text{production}\&\text{degradation}\; \end{array}\right]}}, \end{array}$$ (3)

where *τ*_3_ denotes the average life-time of component X_3_ and $f\left(x_{2}\right)=\lambda x_{2}^{n}/\left(K^{n}+x_{2}^{n}\right)$. For the test examples presented in Fig 2 (dashed blue lines in right panels) we chose *τ*_3_ = 1 and λ = 80, *n* = 2, *K* = 40.

The reaction dynamics of the other variables, i.e., how X_1_, X_2_ affect each other and how they are affected by X_3_ were chosen to achieve diverse system dynamics and are specified in the Materials & Methods. Regardless of the X_1_,X_2_-dynamics, the probability balance equation Eq (2) applied to the specified reaction in Eq (3) imply that the above systems must satisfy the following probability balance relations at stationarity

$$\begin{array}{c} \frac{P(x_{3}=m+1)}{P(x_{3}=m)}=\frac{\left\langle f\left(x_{2}\right)|x_{3}=m\right\rangle }{\left(m+1\right)/\tau_{3}}\;\forall m\in \text{I}\;{\text{N}}_{0}. \end{array}$$ (4)

Although Eq (4) is reminiscent of detailed balance, individual backwards and forward reaction fluxes do not need to balance in general for systems that do not operate at thermodynamic equilibrium such as cellular processes. The condition we exploit here is that the marginal probability distribution does not change at stationarity, and thus that each state must on average balance incoming and outgoing probability fluxes. The contrast with detailed balance is directly apparent in systems with dimeric degradation of X_3_ as discussed in a later section.

As detailed in the Materials & Methods, we employed a numerical algorithm to approximately solve Eq (4) for *f*(*x*_2_) and thus infer how the X_3_ production rate depends on X_2_ from the observed *P*(*x*_2_, *x*_3_). To do so we generated exact realizations of the above stochastic processes using the standard Doob-Gillespie algorithm [26, 27]. We then sampled X_2_, X_3_ from the numerically observed stationary distribution to generate an observed joint probability from *N* = 100, 000 independent samples. Any information about the dynamics of X_1_ was discarded because our method does not utilize any information beyond the pairs of components under consideration. The numerical algorithm is straightforward and detailed in the in the Materials & Methods. In short, a convex optimization algorithm can identify the *f*(*x*_2_) that minimizes Eq (4) for the numerically observed *P*(*x*_2_, *x*_3_). Doing so requires finding a large-dimensional but finite solution vector with elements *f*_*n*_ ≔ *f*(*x*_2_ = *n*) over the observed states that solves a regular least-squares problem as defined in Eq (9). Standard convex optimization allows to find a solution vector constrained to be non-negative and sufficiently smooth by penalizing large second derivative terms as detailed in the Materials & Methods. The results were a near perfect inference for the production rate of X_3_ in all systems as illustrated by the orange crosses in Fig 2. These numerical proof-of-concept examples thus illustrate how the balance equations Eq (4) can be used to reconstruct the functional form of *f*(*x*_2_) from pairwise observation of X_2_, X_3_ in the absence of any temporal information and independent of any information about the vastly different global system dynamics.

Note, in these successful proof-of-principle examples we deliberately made no assumption about *f*(*x*_2_) beyond its smoothness and non-negativity. Alternatively, one could, e.g., assume that *f*(*x*_2_) is a Hill-function and identify its characterizing parameters *K*, *n*, λ through minimizing violations of Eq (4). However, this necessarily requires performing a non-linear optimization with all the drawbacks that entails compared to a linear optimization. If one knows that *f*(*x*_2_) is a Hill-function, one can always fit a Hill-function through the *f*(*x*_2_) obtained from our algorithm and identify *K*, *n*, λ that way.

To fully specify a rate function rather than a finite table of values, e.g., through fitting a Hill function or simply continuing *f*(*x*_2_) = *const*. above and below the largest and smallest state, requires additional *a priori* information. Because such information cannot be deduced from the experimental observations we refrain from doing so throughout the manuscript.

Also note, that when X_2_ varies very slowly *f*(*x*_2_) can be directly read off from the conditional averages 〈*f*(*x*_2_)|*x*_3_〉 as discussed in the next section. The above algorithm solves the problem of determining *f*(*x*_2_) when the timescales of X_2_ and X_3_ are not separable.

### Sampling requirements

Information cannot be created from nothing and the above inference cannot determine rates for states that were never observed. In practice, making additional assumptions to fill in gaps, such as monotonicity or the functional form of *f*(*x*_2_), could prove useful (and are easily incorporated into the algorithm), but here we want to illustrate the core of the inference quality based solely on the convex optimization of Eq (4). We thus define an error heuristic *E* to quantify the quality of our inference by weighting errors in the rate function by the probability of the system to have been observed in that state, relative to the overall average of the rate function 〈*f*_true_〉:

$$\begin{array}{c} E=\sum_{x_{2}}\frac{\left|f_{\text{inferred}}\left(x_{2}\right)-f_{\text{true}}\left(x_{2}\right)\right|}{\left\langle f_{\text{true}}\right\rangle }P\left(x_{2}\right)\;. \end{array}$$ (5)

**Fig 3:**
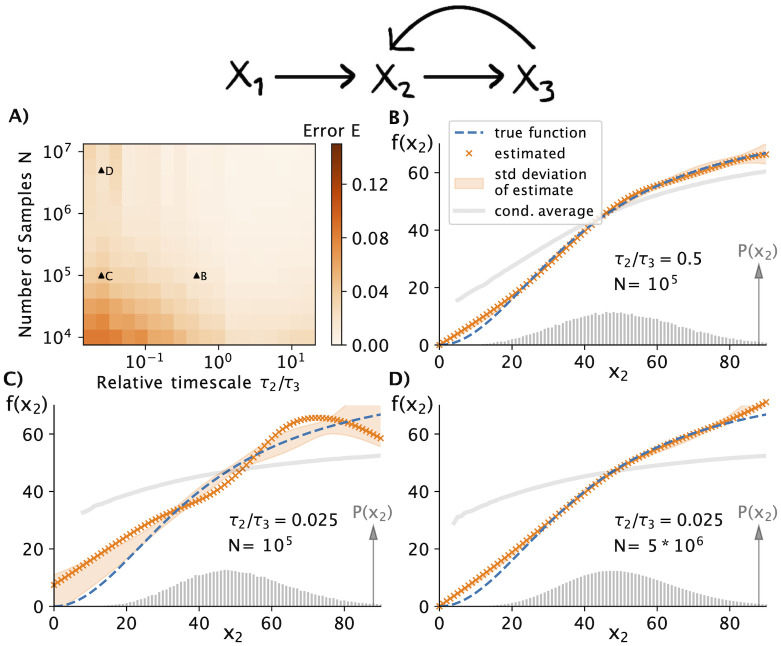
Experimentally achievable sampling leads to accurate inference even when fast upstream variability masks rate dependencies. A) The number of data points required to successfully infer *f*(*x*_2_) from an empirical *P*(*x*_2_, *x*_3_) depends on the relative time-scales between the two components of interest. Simulations of the noise enhancing three-component system (Materials & methods) show that several thousand measurement samples can be enough to reliably infer the rate dependence *f*(*x*_2_) when upstream fluctuations are relatively slow, i.e., *τ*_2_ > *τ*_3_. B) When upstream fluctuations are fast, the downstream variable does not have time to adjust and the conditional average 〈*x*_3_|*x*_2_〉 no longer follows *f*(*x*_2_) as indicated by the grey line. In contrast, our inference method based on Eq (4) accurately estimates the actual rate function from *N* = 100, 000 samples. The orange crosses depict an example of one inference while the shaded area displays the standard deviation of individual inferences from different samples of the same process. C) As the upstream fluctuations in X_2_ become faster, the inference gets worse when using the same number of sampling points. However, even for systems in which X_2_, and X_3_ time-scales are separated 40-fold, the production rate *f*(*x*_2_) can be accurately inferred from *N* = 5 × 10^6^ samples (panel D). Such sampling is experimentally achievable using flow-cytometry approaches to characterize single-cell heterogeneity [28].

To illustrate how the relative time-scale of X_2_ and X_3_ affect this inference error *E* we consider the above “noise enhancing” system (Materials & methods) for which changing lifetimes did not introduce different system dynamics. For such systems, inferring *f*(*x*_2_) is straightforward when the variability of X_2_ is slow such that X_3_ has enough time to adjust to X_2_-levels and the conditional average 〈*x*_3_|*x*_2_〉 directly identifies the production rate of X_3_ (SI). For faster upstream fluctuations the effect of X_2_-variability on X_3_ decreases and the inference of the production rate becomes more challenging. However, compared to the naive statistical approach of interpreting conditional averages as rates, our inference algorithm based on Eq (4) reliably identifies the correct rate function even when the time-scale of X_2_-fluctuations is fast relative to X_3_, see Fig 3B. When the upstream variably becomes more than an order of magnitude faster than X_3_ our inferred rate function deviates significantly from the true one when inferred from a joint probability distribution constructed from *N* = 100, 000 samples. However, even in this unfavourable regime with a 40-fold separation of time-scale between the upstream and downstream variable such that the conditional average 〈*x*_3_|*x*_2_〉 levels-off, *N* = 5 × 10^6^ were enough sample observations to correctly infer *f*(*x*_2_), see Fig 3D.

Note, that the parameter *τ*_3_ is structurally unidentifiable by our approach. However, if it is unknown, our approach will still determine *τ*_3_ ⋅ *f*(*x*_2_), i.e., we can identify the shape of the rate function but not its scale.

### Upregulated *vs.* downregulated production rates

**Fig 4:**
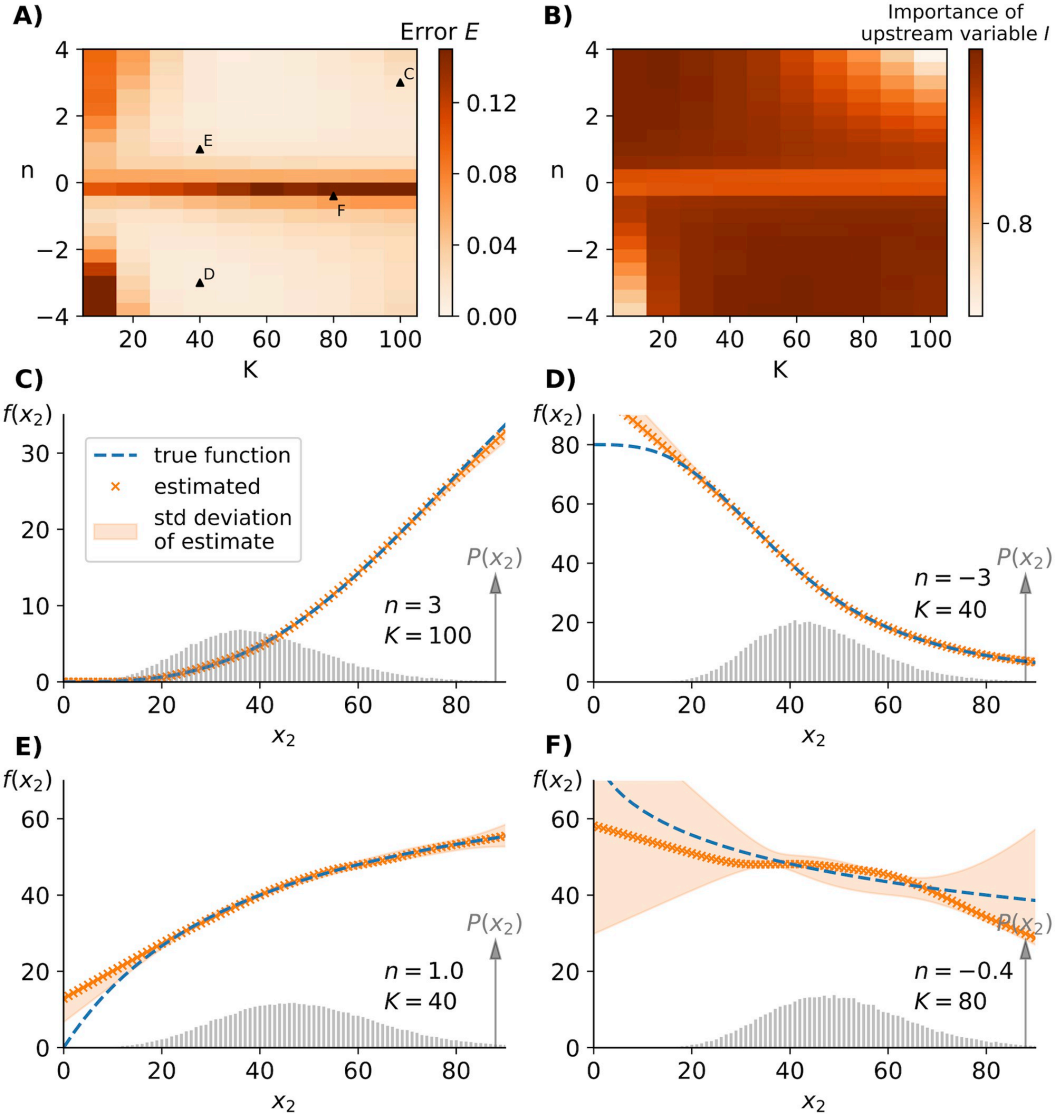
Different shapes of rate functions can be inferred. A) Changing the shape of the production rate $f\left(x_{2}\right)=\lambda x_{2}^{n}/\left(x_{2}^{n}+K^{n}\right)$ by varying *K* and *n* while keeping time-scales fixed and equal, we find that the inferred reaction rates using Eq (4) and *P*(*x*_2_, *x*_3_) agree well with the true rate for *N* = 100, 000 samples across a broad range of the parameter regime. Data shown are for the same noise enhancing three-component system (Materials & methods) as in Fig 3. B) Unsatisfactory inference of *f*(*x*_2_) corresponds to regimes in which the upstream variable has only little influence on the downstream fluctuations as quantified by the relative importance term *I* defined in Eq (6). C,D,E) Successful inference examples for different Hill-functions including repressing effects of X_2_ on X_3_. Any deviations from the true rate function are in the region in which the system is rarely or never observed. F) Example of unsatisfactory inference when the reaction rate varies only little over the majority of observed states resulting in a small effect of X_2_-fluctuations on X_3_-levels. The poor inference is also highlighted by the extremely broad shaded region indicating the standard deviation of inferred *f*(*x*_2_) for identical systems subject to different random sampling.

While the above examples exhibit vastly different global system dynamics the functional form of the production rate of X_3_ was conserved across all systems. Next, we demonstrate that our inference method works for arbitrary Hill-type functions for the production rate. We simulated the noise enhancing three-component system (Materials & methods) from Fig 3 with differently shaped Hill-functions $f\left(x_{2}\right)=\lambda x_{2}^{n}/\left(x_{2}^{n}+K^{n}\right)$ by systematically varying the parameters *K*, *n*. The tested range of parameters reflects biologically relevant different shapes, including negative *n* corresponding to X_2_ suppressing X_3_, small values of *n* such that X_3_ is barely affected by X_2_, as well as strongly cooperative effects with *n* → ±4. As illustrated in Fig 4A, the inference works satisfactorily for *N* = 100, 000 across a broad range Hill-functions with specific examples of successful inference depicted in Fig 4C, 4D and 4E. Note, that those rate functions could not be inferred for states that were never (or extremely rarely) observed.

To determine the cause of unsatisfactory inferences as illustrated by an example in Fig 4F, we utilize the general noise propagation relation [18] to describe all systems within the class of Eq (3)

$$\begin{array}{c} \underset{\eta_{x_{3},x_{3}}}{\underset{︸}{\frac{\text{Var}(x_{3})}{{\left\langle x_{3}\right\rangle }^{2}}}}=\frac{1}{\left\langle x_{3}\right\rangle }+\underset{\eta_{x_{3},f}}{\underset{︸}{\frac{\text{Cov}(x_{3},f\left(x_{2}\right))}{\left\langle x_{3}\right\rangle \left\langle f\left(x_{2}\right)\right\rangle }}}\;,\;I≔\frac{|\eta_{x_{3},f}|}{\eta_{x_{3},x_{3}}}. \end{array}$$ (6)

Here, *I* quantifies how much X_2_-fluctuations affect X_3_-levels. We find that regions of unsatisfactory inference correspond to systems in which the upstream variability has only a small effect on X_3_ (compare panels A and B of Fig 4) Inference in the regime where *n* ≈ 0 can be significantly improved through a simple cross-validation step as discussed in a later section. Alternatively, enforcing additional constrains such as monotonicity can overcome this problem when applicable (SI).

### Non-linear degradation rates

In all of the above systems, X_3_-molecules were degraded in a first-order reaction as is commonly the case for cellular components [29, 30] and would be approximately true for all cellular components that are not actively degraded but effectively diluted by cellular growth [31]. Next, we demonstrate that our inference method works equally well for systems in which X_3_ undergoes non-linear degradation reactions.

**Fig 5:**
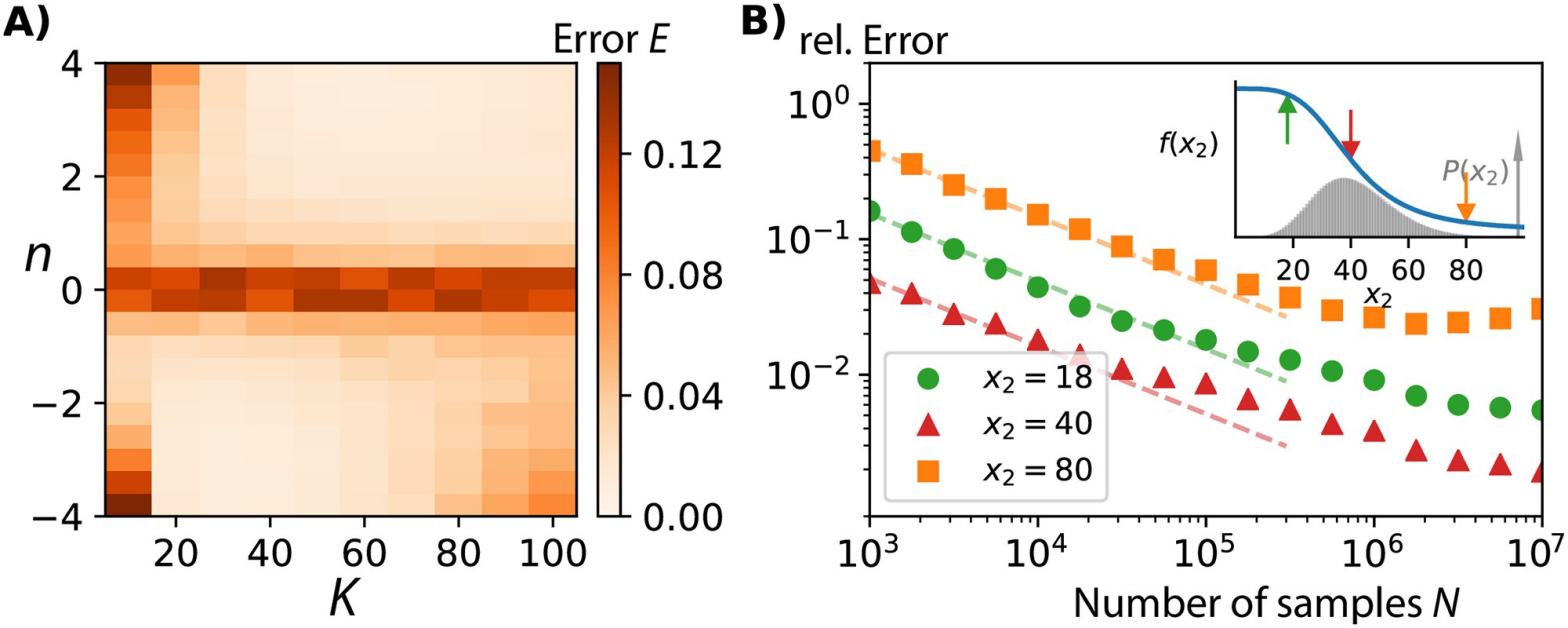
Non-linear degradation does not affect the inference. A) For simulated systems with non-linear degradation of X_3_-molecules as defined in Eq (7), the inference quality is satisfactory when using empirically determined joint probability distributions *P*(*x*_2_, *x*_3_) from *N* = 100, 000 samples. Plotted are the inference error *E* for different production rates $f\left(x_{2}\right)=\lambda x_{2}^{n}/\left(x_{2}^{n}+K^{n}\right)$. Poor inference corresponds to parameter regimes in which the upstream variable X_2_ has only a negligible effect on the downstream variable X_3_, i.e., when *K* or *n* are small. Data are for the same noise enhancing three-component system as in Fig 3 with the only difference that the degradation of X_3_ is now non-linear. B) For a given state, the relative error of the inferred reaction rate *f*(*x*_2_) initially decreases $\propto 1/\sqrt{N}$ (dashed lines) as the number of sampling points *N* increases. However, for large *N*, the relative error levels off, with higher probability states reaching a lower plateau than those only visited rarely. The inset depicts the true function and the specific states considered here, as well as the resulting probability distribution of *x*_2_.

Analogous to Fig 4, we varied the shape of the production rate *f*(*x*_2_) in a class of noise enhancing (Materials & methods) systems with the following conserved part

$$\begin{array}{c} \begin{array}{ccc} x_{3} & \overset{f\left(x_{2}\right)}{\to } & x_{3}+1\\ x_{3} & \overset{\gamma x_{3}\left(x_{3}-1\right)}{\to } & x_{3}-2 \end{array}+\underset{\text{noise}\;\text{enhancing}\;\text{feedback}}{\underset{︸}{\left[\begin{array}{c} {\text{X}}_{1}{,\text{X}}_{2}\\ \text{production}\&\text{degradation} \end{array}\right]}} \end{array}$$ (7)

where a non-linear degradation rate corresponding to a dimerization event was added. We again find that our inference method works reliable for most parameters, see Fig 5A, with unsatisfactory results corresponding again to parameter regimes in which the upstream variable has only a marginal effect on the downstream fluctuations.

Additionally, we analyzed how the inference quality of *f*(*x*_2_) behaves for individual states. As intuitively expected, we find that the inference error initially decreases $\propto 1/\sqrt{N}$ as the number of samples *N* increases. However, for large *N* a plateau becomes apparent that is most severe for the most rarely observed states, Fig 5B. This behaviour was generally observed across different classes of systems (SI). Explicitly accounting for the effects of sampling error may lead to lower plateaus for the estimation errors with more advanced statistical methods to invert Eq (4) but are beyond the scope of the current work.

Note, in these systems the component of interest X_3_ degrades as a dimer, such that its specified degradation rate in Eq (7) is non-linear, and the reaction eliminates two molecules at a time. The probability balancing Eq (2) therefore no longer involves just neighbouring states and detailed balance is broken. Instead, the above systems must satisfy the following balance equations $\forall m\in \text{I}\;{\text{N}}_{0}$

$$\begin{array}{c} \gamma \left(m+1\right)mP(x_{3}=m+1)+\gamma \left(m+2\right)\left(m+1\right)P(x_{3}=m+2)=P(x_{3}=m)\left\langle f\left(x_{2}\right)|x_{3}=m\right\rangle . \end{array}$$ (8)

### Experimental measurement noise

Due to finite sampling and unavoidable measurement noise, empirically observed probability distributions will not perfectly reproduce the stationary distributions of the underlying chemical reaction network. Next, we analyze how measurement noise affects our inference method by explicitly accounting for small absolute and relative error terms as well as systemic undercounting of molecules.

**Fig 6:**
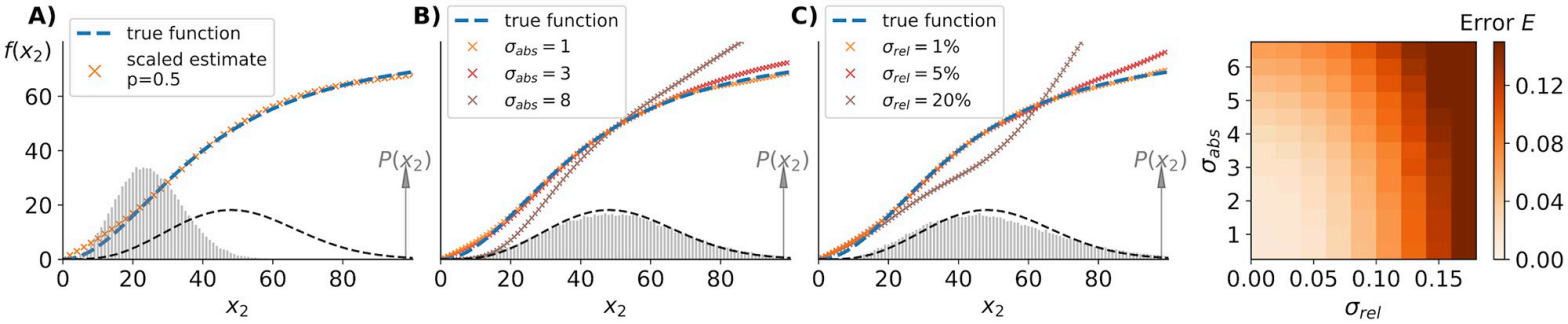
Small measurement noise does not prohibit accurate inference. A) Simulated “empirical” data (histogram) with binomial undercounting in which each molecule is detected with a fixed probability *p*. Our inference algorithm identifies the correct reaction rate if we simply multiply the measured molecule numbers by 1/*p* again to obtain the input function (dashed blue line). If we do not know *p* then the algorithm identifies the correct shape *f*(*x*_2_) but cannot identify the correct scale of X_2_ over which it varies. B) Simulated “empirical” data (grey histogram) with added absolute measurement errors modelled as a two-dimensional Gaussian with zero mean and standard deviation of *σ*_*abs*_ = 8. This distribution is not identical to the theoretical one (dashed black line) and leads to significant deviations in the inference (brown crosses). For smaller absolute errors we observe satisfactory inference, as illustrated by the data for *σ*_*abs*_ = 1, 3. C) Simulating relative measurement errors by multiplying “observed” samples from the exact stationary distribution with a random number from a two-dimensional Gaussian with mean one and standard deviation of *σ*_*rel*_ = 0.01, 0.1, 0.2. For relative errors less than 10% we found satisfactory inference but larger errors led to unacceptable estimates for the production rate *f*(*x*_2_) because of the significant deviation of the “empirical” distribution (grey histogram) from the exact stationary distribution (dashed black line) illustrated for *σ*_*rel*_ = 0.2. Explicit de-convolution steps for known types of measurement noise may significantly improve the inference performance of future algorithms based on Eq (2). D) Quantifying the inference error for absolute and relative measurement errors. Relative measurement errors larger than 10% led to unsatisfactory inference.

To simulate “empirically observed” probability distributions of the above noise enhancing system (Materials & methods) we resampled from the exact stationary distribution *P*(*x*_2_, *x*_3_) while adding a two-dimensional normally distributed error with zero mean and a standard deviation of *σ*_abs_ = 1, 3, 8 molecules respectively (SI). For small absolute errors, we find that the inference method still succeeds to satisfactorily determine the original rate function, see Fig 6B. Note, adding Gaussian noise can lead to negative numbers of molecules. In our analysis of additive noise we discarded those negative “measurements” and re-normalized the resulting distribution.

Furthermore, we analyzed the effect of relative measurement noise by multiplying each sampled data point with a two-dimensional normally distributed error term to simulate a relative error of 1%, 5%, 20% in the observed variables respectively (SI). In its current form, our inference is significantly affected by large multiplicative noise because it causes the “measured” probability distribution to differ significantly from the underlying stationary distribution of the stochastic process, see Fig 6C. Future variants of our inference algorithm may potentially improve on this by performing explicit de-convolution steps [32] to estimate stationary state distributions from experimentally recorded ones before exploiting Eq (2). In fact, measuring error due to probabilistic undercounting can be exactly accounted for by determining the probability *p* to detect a specific molecule, and applying our inference method to the re-scaled probability distribution, see Fig 6A.

### Weakly connected components

Our presented method relies on knowing that one component directly affects the production rate of another. We thus obtained unsatisfactory inferences when the upstream variable barely affects the downstream variable, as illustrated in the regime when *n* → 0 or *K* ≪ 〈*x*_2_〉, see Fig 4A.

**Fig 7:**
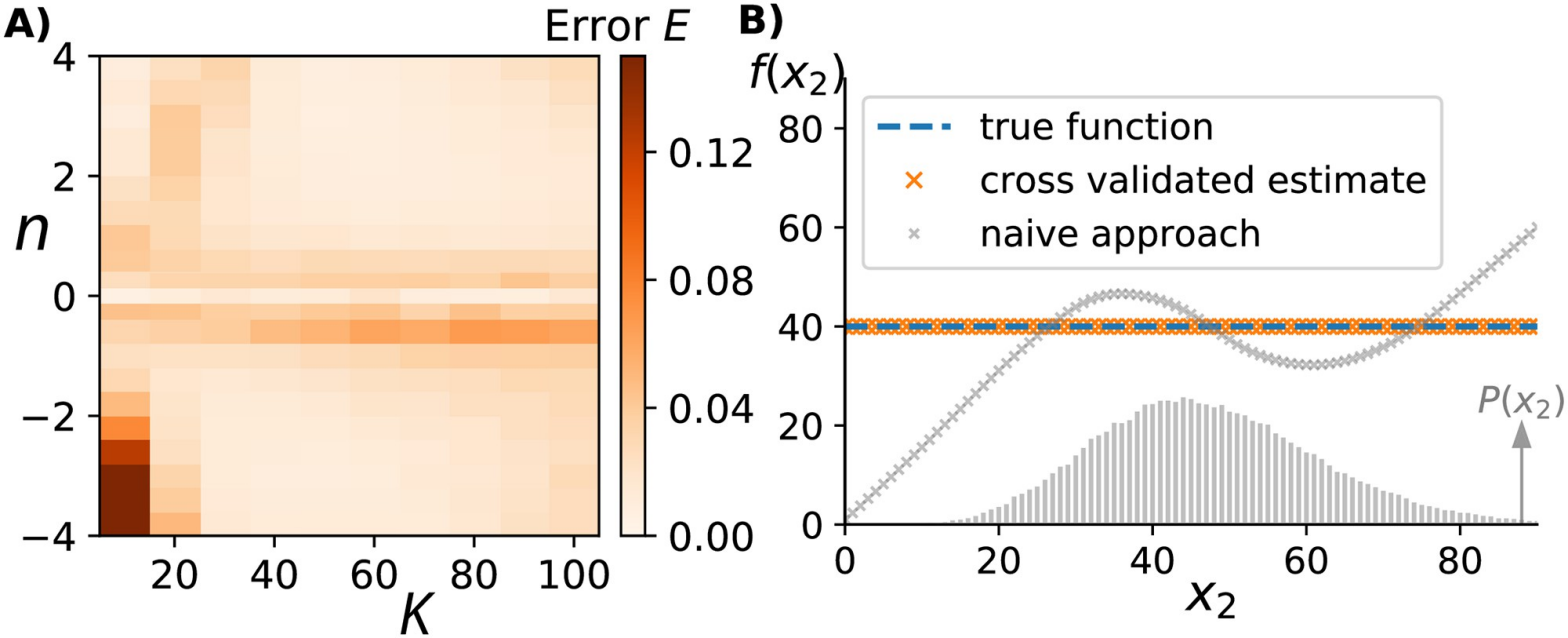
Cross-validation improves inference in regions with constant to near constant rates. A) Analogous to Fig 4 we change the shape of the production rate $f\left(x_{2}\right)=\lambda x_{2}^{n}/\left(x_{2}^{n}+K^{n}\right)$ by varying *K* and *n*. Shown here is the error of the inferred *f*(*x*_2_) when applying our method with an additional cross-validation step as detailed in the Materials & Methods. The quality of the inference is significantly improved in the regime in which the production rate is essentially independent of the upstream variable around *n* ≈ 0. B) Directly comparing the cross-validated and freely inferred *f*(*x*_2_) when applied to a system in which the true production rate was constant. The cross-validated answer picks out the constant production rate, whereas the naive approach gives a varying estimate due to overfitting of Eq (4).

Breakdown of satisfactory inference in that regime can be prevented by explicitly considering the possibility that *f*(*x*_2_) is approximately constant across the observed stochastic fluctuations of X_2_. Following a standard cross-validation approach, we can use one half of the observed data as a “training set” [33]. Using this subset of data we infer *f*(*x*_2_) using our usual unconstrained method but additionally perform a constrained optimization for constant production rates, i.e., we find the best *f*(*x*_2_) = λ for some λ > 0. This by itself will not pick a constant production rate over the freely optimized *f*(*x*_2_) because the latter has many more degrees of freedom. However, because the free optimization overfits sampling errors when minimizing deviations of Eq (4) in the regime in which *f*(*x*_2_) is approximately constant, it will do relatively worse than a constant production rate when applied to the “validation set” of the data. Incorporating this cross-validation approach into our inference methods as detailed in the Materials & Methods, removes the most unsatisfactory regime while leaving the successful inferences unaffected as illustrated in Fig 7A (compare to Fig 4A) with an example detection of constant *f*(*x*_2_) illustrated in Fig 7B.

Identifying constant production rates is a special case of the general problem of identifying which component affects which in complex biochemical reaction networks [34]. Future variants of such a cross-validation approach might thus prove useful in identifying the topology of network interactions based on Eq (4).

### Limitations of the presented evidence

#### Multi-variable dependent rate function

In principle, the basic idea of matching probability fluxes can be applied to components with more than one birth and death reaction as illustrated by the complete generality of Eq (2) which makes no assumption about the number of production and degradation reactions for the species of interest. However, in our proof-of-principle examples we successfully specifically inferred univariable rate functions *f* = *f*(*x*_2_). Whether our algorithm can also successfully identify more complex rate functions such as *f* = *f*(*x*_2_, *x*_1_) will require future work.

Importantly, the value of our approach lies not necessarily in identifying arbitrarily complex rate functions but in identifying simple rate functions in arbitrarily complex systems. The benefit of our approach lies in the fact that it effectively “eliminates” network complexity when reconstructing simple rate laws for local interactions between variables. Note, many complex biological control systems indeed involve many simple interactions which in turn lead to complex systems through feedback loops and an overall large number of interacting components [35].

#### Experimental accuracy

As presented, our method requires highly accurate and discrete probability distributions which is motivated by measuring small numbers of molecules in cells where such discreteness is fundamentally built into the problem and single-molecule accuracy is possible [36–40].

This type of data is at the forefront of what is technologically possible [41, 42] and many current real-life experimental methods will be much less accurate than our simulated input distributions. We have partially addressed this issue by analyzing the effect of experimental errors in the form of Gaussian noise and probabilistic undercounting, see Fig 6. However in real-world experiments, the discreteness of measurements may become completely washed out and sampling might not be as high as in our input distributions obtained from 100,000 data points over around 100 discrete states. Generalizing our approach to continuous distributions and more sparsely sampled distributions with significant sampling error is left for future work.

#### Growing and dividing cells

As is, our method cannot be directly applied to experimental data from growing and dividing cells. Future work is required to demonstrate that our approach could potentially be exploited to infer production rates of components from single-cell data.

In the simplest case, biochemical reactions in growing and dividing cells can be modelled as cyclo-stationary processes of periodically changing probability distributions [43]. In principle, Eq (2) can be generalized to time-averages of time-dependent distributions (see supplement of [18]) but all our proof-of-principle examples considered a stationary component of interest undergoing first or second order degradation in the absence of cell divison. In contrast, much recent progress has been made to analyze stochastic biochemical kinetics in cellular models that include cell-division, gene replication, growth dependent effects, and variability in cell-division times [44–49]. Future work should bring together the approach developed in this manuscript with the existing work on modelling cell-cycle effects.

Additionally, in growing populations time-averaged ensemble averages do not agree with population averages because the former corresponds to a uniform cell-age distribution whereas in a growing population we must have twice as many newborn cells as dividing cells which leads to a non-uniform age-structure [50]. As presented, our evidence corresponds to cellular populations of constant size. Future work is required to show the same approach works in populations with growing number of cells, which in principle can be related to the time-averages in the cyclo-stationary version of Eq (2) as long as the age-structure of cells in the population is known [25].

Formulating stochastic models in terms of concentrations rather than abundances might partially resolve these issues because on average concentrations are the same at the beginning and the end of the cell-cycle unlike abundances which must double on average.

## Discussion

Early aeronautical engineering faced a challenge analogous to that of current synthetic biology. While the qualitative requirements for motored flight were well known, designing a reliable flyer required a breakthrough in *quantitatively* understanding each subproblem through painstaking experimental measurements [51].

Similarly, designing reliable synthetic circuits in biology requires a quantitative description of biochemical reaction dynamics rather than qualitative network interaction models. Here, we present a method that promises to iteratively turn a qualitative model of biochemical interactions into a network of quantitative reaction rates. Given one arrow within a network of interacting components, our method can identify the functional dependence of the actual reaction rate, one interaction at a time, from fluctuations of a subset of components without having to perturb the system.

Existing mechanistic modelling approaches often rely on temporal data [52–59] and generally have to be identified from data in one fell swoop [60–65] with all the reliability issues that come with optimizing over many degrees of freedom at once. Prior work has established approaches that can infer a small number of parameters from perturbation observations of a subset of components in otherwise completely specified multivariable models that were assumed to be a complete and correct representation of the process of interest [19, 66–68]. Some work specifically determines instantaneous production rates from static snapshots of partially observed networks [69], but these previous mechanistic approaches estimate rates and parameters by assuming (near) complete knowledge of the entire network dynamics whereas our approach does not rely on such information. Instead we leave all dynamics unspecified that do not directly affect the component of interest.

While approaches that combine mechanistic models with Bayesian inference [70–73] have been used to account for significant noise in experimental data, they too rely on inferring parameters for fully defined mechanistic models all at once. Statistical approaches that rely on perturbations [7–11] can be straightforwardly applied to static snapshots of incompletely observed complex systems but fail to account for the dynamic ways in which one component affects another, and thus do generally not quantitatively describe physical interactions between components.

Our results show that we can reliably identify reaction rates independent of the larger network structures, in contrast to previous approaches in which small models of gene expression were inverted under the assumption they were correct as a whole and only a handful of parameter values needed to be determined [36, 74, 75].

The presented numerical proof-of-principle results establish that our presented algorithm works across a broad range of tested systems and that the number of required observations for algorithmic success is comparable to those accessible by modern experimental single-cell techniques in biology such as flow-cytometry that routinely measure millions of isogenic cells at a time.

As presented, our algorithm requires highly accurate input distributions, and we cannot exclude the possibility that our method works only in theory but not in practice. However, here we presented only the most basic inference algorithm to extract information from the probability flux balance relations. We thereby established that the information contained in Eq (2) is enough to reconstruct the shape of biochemical rate functions in principle. Hybrid approaches that, e.g., combine statistical tools and neural networks with first-principle mathematical modelling have been successfully used to identify parameters in biochemical reaction network [19, 76]. We thus expect future work that combines advanced statistical methods with our first-principle approach of extracting information from Eq (2) to outperform our naive algorithm in the face of significant sampling or measurement error. Additionally, more general approaches of utilizing Eq (2) may in the future be able to identify the network topology within interaction networks from static snapshots of joint probability distributions. This is a problem of immense current interest in systems biology, e.g., for detecting causal interactions from static snapshots of single-cell RNA-seq data.

## Materials & methods

### Basic algorithm

To utilize probability distributions that contain sampling errors we write the balance Eq (4) for our example systems as *G***f** = **h**, where *G*_*ij*_ = *P*(*x*_2_ = *j*, *x*_3_ = *i*), *f*_*i*_ = *f*(*x*_2_ = *i*) and *h*_*i*_ = (*i* + 1)*P*(*x*_3_ = *i* + 1)/*τ*_3_. To avoid overfitting the solution **f** to sampling noise we add a regularization term that effectively penalizes the discrete “second derivatives” *f*_*i*+2_ − 2*f*_*i*+1_ + *f*_*i*_. The effect of this regularization is to smoothen the resulting reaction rate function. Motivated by complex cellular processes where *f*(*x*_2_) represent biochemical reaction rates, we furthermore constrain the solution vector **f** to be non-negative. We thus solve the following optimization problem

$$\begin{array}{c} \underset{f}{\text{min}}\;\{||Gf-{h||}^{2}+{\epsilon ||\Gamma f||}^{2}\}\;s.t.\;f\geq 0 \end{array}$$ (9)

where Γ_*ij*_ = *δ*_*i*,*j*_ − 2*δ*_*i*,*j*+1_ + *δ*_*i*,*j*+2_ is the regularization matrix and *ϵ* corresponds to the strength of the smoothening. The strength of this regularization parameter affects the quality of the inference. We found $\epsilon =1/\sqrt{N}$ to lead to satisfactory results because it appropriately decreases in strength as the number of sampling points *N* increases. This regularization was used throughout the paper. In other applications, alternative heuristics to choose *ϵ* may prove useful to achieve satisfactory results (SI). Note, that if the lifetime *τ*_3_ is unknown, the inference method will still correctly identify *τ*_3_ ⋅ *f*(*x*_2_) meaning that we get the correct shape of the reaction rate function up to an unknown scale-factor.

We solved Eq (9) using a standard convex optimization approach which is guaranteed to converge to the optimal solution as a linear program [77]. The code is provided at https://github.com/t-wittenstein/quantify-biochemical-rates. While it is straightforward to add further constraints, such as monotonicity, about the solution function *f*(*x*_2_) we here deliberately only present results without making any additional assumptions beyond Eq (9). Alternatively one could utilize additional information about *f*(*x*_2_) and, say, fit a Hill-function directly by minimizing Eq (9). However, this necessarily involves a non-linear optimization routine to find the best-fit parameters *K*, *n*, λ which is in general less robust than the linear optimization problem we solve above.

### Cross-validation algorithm

In order to avoid overfitting systems when production rates are approximately constant we compared our inferred production rate against a constant one as follows: We divided the sampling data into two equally sized sets, and applied our inference method first to the “training” set and then checked against a constant production rate using the second “validation” set [33]. To compare the two rates we calculated the violation of Eq (4) as the sum of all squared errors. If the constant production rate’s error was smaller or within a 5% margin of the freely fitted production rate from the training set, we determined a constant production rate to be the best inference and optimized it over the whole data. Otherwise we applied our regular inference method to the full data set instead.

### Definition of example systems

We considered simple three-component systems in which the production and degradation rates of X_1_,X_2_,X_2_ took the following form

$$\begin{array}{c} \begin{array}{ccc} x_{i}\; & \overset{f_{i}\left(x\right)}{\to } & \;x_{i}+1\\ x_{i}\; & \overset{x_{i}/\tau_{i}}{\to } & \;x_{i}-1 \end{array}. \end{array}$$ (10)

In particular, we simulated the following example systems depicted in Fig 2 where the production and degradation rate for X_3_ was kept the same as specified in the main text while the other components were subject to the following dynamics.

- Bistable system: $f_{1}\left(x_{2}\right)=\lambda x_{2}^{n}/\left(K^{n}+x_{2}^{n}\right)+c,f_{2}\left(x_{1}\right)=x_{1}$ with λ = 50, *n* = 6, *K* = 37, *c* = 15 and life-times *τ*_1_ = *τ*_2_ = 1.
- Oscillating system: $f_{1}\left(x_{3}\right)=\lambda_{1}K_{1}^{n_{1}}/\left(K_{1}^{n_{1}}+x_{3}^{n_{1}}\right)$, $f_{2}\left(x_{1}\right)=\lambda_{2}x_{1}^{n_{2}}/\left(K_{2}^{n_{2}}+x_{1}^{n_{2}}\right)$ with λ_1_ = 50000, *n*_1_ = 10, *K*_1_ = 0.1, λ_2_ = 80, *n*_2_ = 1, *K*_2_ = 100 and life-times *τ*_1_ = *τ*_2_ = 1.
- Noise controlling system: *f*_1_(*x*_3_) = λ_1_ *x*_3_, $f_{2}\left(x_{1}\right)=\lambda_{2}K_{2}^{n_{2}}/\left(K_{2}^{n_{2}}+x_{1}^{n_{2}}\right)$ with λ_1_ = 50, λ_2_ = 3000, *n*_2_ = 10, *K*_2_ = 10 and life-times *τ*_1_ = 50, *τ*_2_ = 1.
- Noise enhancing system: *f*_1_ = 5, $f_{2}\left(x_{1},x_{3}\right)=\;\lambda x_{3}^{n}/\left(K^{n}+x_{3}^{n}\right)+cx_{1}$ with λ = 25, *n* = 4, *K* = 50, *c* = 8 and life-times *τ*_1_ = *τ*_2_ = 1.

Data in Figs 3–7 correspond to the above noise enhancing system with modifications as specified in the main text. The systems with non-linear degradation rate *γx*_3_(*x*_3_ − 1) were simulated with *γ* = 2.

## Supporting information

S1 Text Supplementary Information of the paper: “Quantifying biochemical reaction rates from static population variability within incompletely observed complex networks”. Detailed description of simulation process, models, and supplementary data.
(PDF)

## References

[1] Islam S, Kjällquist U, Moliner A, Zajac P, Fan JB, Lönnerberg P, et al. Characterization of the single-cell transcriptional landscape by highly multiplex RNA-seq. Genome research. 2011;21(7):1160–1167. doi: 10.1101/gr.110882.110 21543516 PMC3129258

[2] Svensson V, Vento-Tormo R, Teichmann SA. Exponential scaling of single-cell RNA-seq in the past decade. Nature Protocols. 2018;13(4):599–604. doi: 10.1038/nprot.2017.149 29494575

[3] Robinson JP, Roederer M. Flow cytometry strikes gold. Science. 2015;350(6262):739–740. 26564833 10.1126/science.aad6770

[4] Zlokarnik G, Negulescu PA, Knapp TE, Mere L, Burres N, Feng L, et al. Quantitation of transcription and clonal selection of single living cells with *β*-lactamase as reporter. Science. 1998;279(5347):84–88. doi: 10.1126/science.279.5347.84 9417030

[5] Porichis F, Hart MG, Griesbeck M, Everett HL, Hassan M, Baxter AE, et al. High-throughput detection of miRNAs and gene-specific mRNA at the single-cell level by flow cytometry. Nature Communications. 2014;5(1):1–12. doi: 10.1038/ncomms6641 25472703 PMC4256720

[6] Jaitin DA, Kenigsberg E, Keren-Shaul H, Elefant N, Paul F, Zaretsky I, et al. Massively parallel single-cell RNA-seq for marker-free decomposition of tissues into cell types. Science. 2014;343(6172):776–779. doi: 10.1126/science.1247651 24531970 PMC4412462

[7] Rizvi AH, Camara PG, Kandror EK, Roberts TJ, Schieren I, Maniatis T, et al. Single-cell topological RNA-seq analysis reveals insights into cellular differentiation and development. Nature Biotechnology. 2017;35(6):551. doi: 10.1038/nbt.3854 28459448 PMC5569300

[8] Chen R, Wu X, Jiang L, Zhang Y. Single-cell RNA-seq reveals hypothalamic cell diversity. Cell Reports. 2017;18(13):3227–3241. doi: 10.1016/j.celrep.2017.03.004 28355573 PMC5782816

[9] Tirosh I, Venteicher AS, Hebert C, Escalante LE, Patel AP, Yizhak K, et al. Single-cell RNA-seq supports a developmental hierarchy in human oligodendroglioma. Nature. 2016;539(7628):309–313. doi: 10.1038/nature20123 27806376 PMC5465819

[10] Filbin MG, Tirosh I, Hovestadt V, Shaw ML, Escalante LE, Mathewson ND, et al. Developmental and oncogenic programs in H3K27M gliomas dissected by single-cell RNA-seq. Science. 2018;360(6386):331–335. doi: 10.1126/science.aao4750 29674595 PMC5949869

[11] Risso D, Perraudeau F, Gribkova S, Dudoit S, Vert JP. A general and flexible method for signal extraction from single-cell RNA-seq data. Nature Communications. 2018;9(1):1–17. doi: 10.1038/s41467-017-02554-5 29348443 PMC5773593

[12] Liu F, Zhang SW, Guo WF, Wei ZG, Chen L. Inference of gene regulatory network based on local Bayesian networks. PLoS computational biology. 2016;12(8):e1005024. doi: 10.1371/journal.pcbi.1005024 27479082 PMC4968793

[13] Weistuch C, Agozzino L, Mujica-Parodi LR, Dill KA. Inferring a network from dynamical signals at its nodes. PLoS computational biology. 2020;16(11):e1008435. doi: 10.1371/journal.pcbi.1008435 33253160 PMC7728228

[14] Mayer J, Khairy K, Howard J. Drawing an elephant with four complex parameters. American Journal of Physics. 2010;78(6):648–649. doi: 10.1119/1.3254017

[15] Pearl J, et al. Causal inference in statistics: An overview. Statistics surveys. 2009;3:96–146. doi: 10.1214/09-SS057

[16] Millstein J, Zhang B, Zhu J, Schadt EE. Disentangling molecular relationships with a causal inference test. BMC Genetics. 2009;10(1):23. doi: 10.1186/1471-2156-10-23 19473544 PMC3224661

[17] Schadt EE, Lamb J, Yang X, Zhu J, Edwards S, GuhaThakurta D, et al. An integrative genomics approach to infer causal associations between gene expression and disease. Nature Genetics. 2005;37(7):710–717. doi: 10.1038/ng1589 15965475 PMC2841396

[18] Hilfinger A, Norman TM, Vinnicombe G, Paulsson J. Constraints on Fluctuations in Sparsely Characterized Biological Systems. Phys Rev Lett. 2016;116:058101. doi: 10.1103/PhysRevLett.116.058101 26894735 PMC4834202

[19] Lee D, Jayaraman A, Kwon JS. Development of a hybrid model for a partially known intracellular signaling pathway through correction term estimation and neural network modeling. PLoS Computational Biology. 2020;16(12):e1008472. doi: 10.1371/journal.pcbi.1008472 33315899 PMC7769624

[20] Haggerty RA, Purvis JE. Inferring the structures of signaling motifs from paired dynamic traces of single cells. PLoS computational biology. 2021;17(2):e1008657. doi: 10.1371/journal.pcbi.1008657 33539338 PMC7889133

[21] Grima R. A study of the accuracy of moment-closure approximations for stochastic chemical kinetics. The Journal of chemical physics. 2012;136(15):04B616. doi: 10.1063/1.3702848 22519313

[22] Lakatos E, Ale A, Kirk PD, Stumpf MP. Multivariate moment closure techniques for stochastic kinetic models. The Journal of chemical physics. 2015;143(9):094107. doi: 10.1063/1.4929837 26342359

[23] Mazo RM. On the discrepancy between results of Nicolis and Saito concerning fluctuations in chemical reactions. The Journal of Chemical Physics. 1975;62(10):4244–4244. doi: 10.1063/1.430277

[24] Kelly FP. Reversibility and stochastic networks. Cambridge University Press; 2011.

[25] Thomas P. Making sense of snapshot data: ergodic principle for clonal cell populations. Journal of The Royal Society Interface. 2017;14(136):20170467. doi: 10.1098/rsif.2017.0467 29187636 PMC5721154

[26] Doob JL. Markoff Chains–Denumerable Case. Transactions of the American Mathematical Society. 1945;58(3):455–473.

[27] Gillespie DT. A general method for numerically simulating the stochastic time evolution of coupled chemical reactions. Journal of Computational Physics. 1976;22(4):403–434. doi: 10.1016/0021-9991(76)90041-3

[28] Arrigucci R, Bushkin Y, Radford F, Lakehal K, Vir P, Pine R, et al. FISH-Flow, a protocol for the concurrent detection of mRNA and protein in single cells using fluorescence in situ hybridization and flow cytometry. Nature Protocols. 2017;12(6):1245–1260. doi: 10.1038/nprot.2017.039 28518171 PMC5548662

[29] Wang Y, Liu CL, Storey JD, Tibshirani RJ, Herschlag D, Brown PO. Precision and functional specificity in mRNA decay. Proceedings of the National Academy of Sciences. 2002;99(9):5860–5865. doi: 10.1073/pnas.092538799 11972065 PMC122867

[30] Belle A, Tanay A, Bitincka L, Shamir R, O’Shea EK. Quantification of protein half-lives in the budding yeast proteome. Proceedings of the National Academy of Sciences. 2006;103(35):13004–13009. doi: 10.1073/pnas.0605420103 16916930 PMC1550773

[31] Huh D, Paulsson J. Non-genetic heterogeneity from stochastic partitioning at cell division. Nature Genetics. 2011;43(2):95–100. doi: 10.1038/ng.729 21186354 PMC3208402

[32] Eraslan G, Simon LM, Mircea M, Mueller NS, Theis FJ. Single-cell RNA-seq denoising using a deep count autoencoder. Nature Communications. 2019;10(1):1–14. doi: 10.1038/s41467-018-07931-2 30674886 PMC6344535

[33] Shao J. Linear model selection by cross-validation. Journal of the American statistical Association. 1993;88(422):486–494. doi: 10.1080/01621459.1993.10476299

[34] Mercatelli D, Scalambra L, Triboli L, Ray F, Giorgi FM. Gene regulatory network inference resources: a practical overview. Biochimica et Biophysica Acta (BBA)-Gene Regulatory Mechanisms. 2020;1863(6):194430. doi: 10.1016/j.bbagrm.2019.194430 31678629

[35] Milo R, Shen-Orr S, Itzkovitz S, Kashtan N, Chklovskii D, Alon U. Network motifs: simple building blocks of complex networks. Science. 2002;298(5594):824–827. doi: 10.1126/science.298.5594.824 12399590

[36] Taniguchi Y, Choi PJ, Li GW, Chen H, Babu M, Hearn J, et al. Quantifying E. coli proteome and transcriptome with single-molecule sensitivity in single cells. Science. 2010;329(5991):533–538. doi: 10.1126/science.1188308 20671182 PMC2922915

[37] Okumus B, Landgraf D, Lai GC, Bakshi S, Arias-Castro JC, Yildiz S, et al. Mechanical slowing-down of cytoplasmic diffusion allows in vivo counting of proteins in individual cells. Nature communications. 2016;7(1):1–11. doi: 10.1038/ncomms11641 PMC4873973 27189321

[38] Uphoff S, Lord ND, Okumus B, Potvin-Trottier L, Sherratt DJ, Paulsson J. Stochastic activation of a DNA damage response causes cell-to-cell mutation rate variation. Science. 2016;351(6277):1094–1097. doi: 10.1126/science.aac9786 26941321 PMC4827329

[39] Sepúlveda LA, Xu H, Zhang J, Wang M, Golding I. Measurement of gene regulation in individual cells reveals rapid switching between promoter states. Science. 2016;351(6278):1218–1222. doi: 10.1126/science.aad0635 26965629 PMC4806797

[40] Lepore A, Taylor H, Landgraf D, Okumus B, Jaramillo-Riveri S, McLaren L, et al. Quantification of very low-abundant proteins in bacteria using the HaloTag and epi-fluorescence microscopy. Scientific reports. 2019;9(1):1–9. doi: 10.1038/s41598-019-44278-0 31133640 PMC6536506

[41] Elf J, Barkefors I. Single-molecule kinetics in living cells. Annual review of biochemistry. 2019;88:635–659. doi: 10.1146/annurev-biochem-013118-110801 30359080

[42] Hardo G, Bakshi S. Challenges of analysing stochastic gene expression in bacteria using single-cell time-lapse experiments. Essays in Biochemistry. 2021;65(1):67–79. doi: 10.1042/EBC20200015 33835126 PMC8056041

[43] Huh D, Paulsson J. Random partitioning of molecules at cell division. Proc Natl Acad Sci U S A. 2011;108(36):15004–15009. doi: 10.1073/pnas.1013171108 21873252 PMC3169110

[44] Cao Z, Grima R. Analytical distributions for detailed models of stochastic gene expression in eukaryotic cells. Proceedings of the National Academy of Sciences. 2020;117(9):4682–4692. doi: 10.1073/pnas.1910888117 32071224 PMC7060679

[45] Beentjes CH, Perez-Carrasco R, Grima R. Exact solution of stochastic gene expression models with bursting, cell cycle and replication dynamics. Physical Review E. 2020;101(3):032403. doi: 10.1103/PhysRevE.101.032403 32290003

[46] Jędrak J, Kwiatkowski M, Ochab-Marcinek A. Exactly solvable model of gene expression in a proliferating bacterial cell population with stochastic protein bursts and protein partitioning. Physical Review E. 2019;99(4):042416. doi: 10.1103/PhysRevE.99.042416 31108597

[47] Thomas P, Shahrezaei V. Coordination of gene expression noise with cell size: analytical results for agent-based models of growing cell populations. Journal of the Royal Society Interface. 2021;18(178):20210274. doi: 10.1098/rsif.2021.0274 34034535 PMC8150024

[48] Jia C, Grima R. Frequency domain analysis of fluctuations of mRNA and protein copy numbers within a cell lineage: theory and experimental validation. Physical Review X. 2021;11(2):021032. doi: 10.1103/PhysRevX.11.021032

[49] Jędrak J, Ochab-Marcinek A. Contributions to the ‘noise floor’ in gene expression in a population of dividing cells. Scientific Reports. 2020;10(1):1–13. 32782314 10.1038/s41598-020-69217-2 PMC7419568

[50] Powell E. Growth rate and generation time of bacteria, with special reference to continuous culture. Microbiology. 1956;15(3):492–511. 13385433 10.1099/00221287-15-3-492

[51] Jakab PL. Visions of a flying machine: The Wright brothers and the process of invention. Smithsonian Institution; 2014.

[52] Reinker S, Altman RM, Timmer J. Parameter estimation in stochastic biochemical reactions. IEE Proceedings-Systems Biology. 2006;153(4):168–178. doi: 10.1049/ip-syb:20050105 16986618

[53] Tian T, Xu S, Gao J, Burrage K. Simulated maximum likelihood method for estimating kinetic rates in gene expression. Bioinformatics. 2007;23(1):84–91. doi: 10.1093/bioinformatics/btl552 17068087

[54] Fröhlich F, Kaltenbacher B, Theis FJ, Hasenauer J. Scalable parameter estimation for genome-scale biochemical reaction networks. PLoS Computational Biology. 2017;13(1):e1005331. doi: 10.1371/journal.pcbi.1005331 28114351 PMC5256869

[55] Feng Xj, Rabitz H. Optimal identification of biochemical reaction networks. Biophysical Journal. 2004;86(3):1270–1281. doi: 10.1016/S0006-3495(04)74201-0 14990460 PMC1303968

[56] Schmidt H, Cho KH, Jacobsen EW. Identification of small scale biochemical networks based on general type system perturbations. The FEBS Journal. 2005;272(9):2141–2151. doi: 10.1111/j.1742-4658.2005.04605.x 15853799

[57] Ruttor A, Opper M. Efficient statistical inference for stochastic reaction processes. Physical Review Letters. 2009;103(23):230601. doi: 10.1103/PhysRevLett.103.230601 20366136

[58] Kim J, Bates DG, Postlethwaite I, Heslop-Harrison P, Cho KH. Least-squares methods for identifying biochemical regulatory networks from noisy measurements. BMC Bioinformatics. 2007;8(1):1–15. doi: 10.1186/1471-2105-8-8 17212835 PMC1793997

[59] Kitayama T, Kinoshita A, Sugimoto M, Nakayama Y, Tomita M. A simplified method for power-law modelling of metabolic pathways from time-course data and steady-state flux profiles. Theoretical Biology and Medical Modelling. 2006;3(1):1–9. doi: 10.1186/1742-4682-3-24 16846504 PMC1550393

[60] Ashyraliyev M, Fomekong-Nanfack Y, Kaandorp JA, Blom JG. Systems biology: parameter estimation for biochemical models. The FEBS journal. 2009;276(4):886–902. doi: 10.1111/j.1742-4658.2008.06844.x 19215296

[61] Mendes P, Kell D. Non-linear optimization of biochemical pathways: applications to metabolic engineering and parameter estimation. Bioinformatics (Oxford, England). 1998;14(10):869–883. doi: 10.1093/bioinformatics/14.10.869 9927716

[62] Rodriguez-Fernandez M, Mendes P, Banga JR. A hybrid approach for efficient and robust parameter estimation in biochemical pathways. Biosystems. 2006;83(2-3):248–265. doi: 10.1016/j.biosystems.2005.06.016 16236429

[63] Ocone A, Haghverdi L, Mueller NS, Theis FJ. Reconstructing gene regulatory dynamics from high-dimensional single-cell snapshot data. Bioinformatics. 2015;31(12):i89–i96. doi: 10.1093/bioinformatics/btv257 26072513 PMC4765871

[64] Stathopoulos V, Girolami MA. Markov chain Monte Carlo inference for Markov jump processes via the linear noise approximation. Philosophical Transactions of the Royal Society A: Mathematical, Physical and Engineering Sciences. 2013;371(1984):20110541. doi: 10.1098/rsta.2011.0541 23277599

[65] Dixit PD, Jain A, Stock G, Dill KA. Inferring transition rates of networks from populations in continuous-time Markov processes. Journal of chemical theory and computation. 2015;11(11):5464–5472. doi: 10.1021/acs.jctc.5b00537 26574334

[66] Hasenauer J, Waldherr S, Doszczak M, Radde N, Scheurich P, Allgöwer F. Identification of models of heterogeneous cell populations from population snapshot data. BMC bioinformatics. 2011;12(1):1–15. doi: 10.1186/1471-2105-12-125 21527025 PMC3114742

[67] Hasenauer J, Waldherr S, Wagner K, Allgöwer F. Parameter identification, experimental design and model falsification for biological network models using semidefinite programming. IET systems biology. 2010;4(2):119–130. doi: 10.1049/iet-syb.2009.0030 20232992

[68] Lee D, Jayaraman A, Kwon JSI. Identification of a time-varying intracellular signalling model through data clustering and parameter selection: application to NF-*κ*B signalling pathway induced by LPS in the presence of BFA. IET systems biology. 2019;13(4):169–179. doi: 10.1049/iet-syb.2018.5079 31318334 PMC8687386

[69] La Manno G, Soldatov R, Zeisel A, Braun E, Hochgerner H, Petukhov V, et al. RNA velocity of single cells. Nature. 2018;560(7719):494–498. doi: 10.1038/s41586-018-0414-6 30089906 PMC6130801

[70] Liepe J, Kirk P, Filippi S, Toni T, Barnes CP, Stumpf MP. A framework for parameter estimation and model selection from experimental data in systems biology using approximate Bayesian computation. Nature Protocols. 2014;9(2):439–456. doi: 10.1038/nprot.2014.025 24457334 PMC5081097

[71] Boys RJ, Wilkinson DJ, Kirkwood TB. Bayesian inference for a discretely observed stochastic kinetic model. Statistics and Computing. 2008;18(2):125–135. doi: 10.1007/s11222-007-9043-x

[72] Golightly A, Wilkinson DJ. Bayesian parameter inference for stochastic biochemical network models using particle Markov chain Monte Carlo. Interface Focus. 2011;1(6):807–820. doi: 10.1098/rsfs.2011.0047 23226583 PMC3262293

[73] Zechner C, Pelet S, Peter M, Koeppl H. Recursive Bayesian estimation of stochastic rate constants from heterogeneous cell populations. In: 2011 50th IEEE Conference on Decision and Control and European Control Conference. IEEE; 2011. p. 5837–5843.

[74] Cai L, Friedman N, Xie XS. Stochastic protein expression in individual cells at the single molecule level. Nature. 2006;440(7082):358–362. doi: 10.1038/nature04599 16541077

[75] Friedman N, Cai L, Xie XS. Linking stochastic dynamics to population distribution: an analytical framework of gene expression. Physical Review Letters. 2006;97(16):168302. doi: 10.1103/PhysRevLett.97.168302 17155441

[76] Lee D, Jayaraman A, Kwon JSI. Identification of cell-to-cell heterogeneity through systems engineering approaches. AIChE Journal. 2020;66(5):e16925. doi: 10.1002/aic.16925

[77] Boyd S, Boyd SP, Vandenberghe L. Convex optimization. Cambridge University Press; 2004.

